# Speakers are more cooperative and less individual when interacting in larger group sizes

**DOI:** 10.3389/fpsyg.2023.1145572

**Published:** 2023-06-05

**Authors:** Elisa Pellegrino, Volker Dellwo

**Affiliations:** Department of Computational Linguistics, University of Zurich, Zurich, Switzerland

**Keywords:** group size, cooperation, vocal accommodation, voice individualization, automatic speaker recognition

## Abstract

**Introduction:**

Cooperation, acoustically signaled through vocal convergence, is facilitated when group members are more similar. Excessive vocal convergence may, however, weaken individual recognizability. This study aimed to explore whether constraints to convergence can arise in circumstances where interlocutors need to enhance their vocal individuality. Therefore, we tested the effects of group size (3 and 5 interactants) on vocal convergence and individualization in a social communication scenario in which individual recognition by voice is at stake.

**Methods:**

In an interactive game, players had to recognize each other through their voices while solving a cooperative task online. The vocal similarity was quantified through similarities in speaker i-vectors obtained through probabilistic linear discriminant analysis (PLDA). Speaker recognition performance was measured through the system Equal Error Rate (EER).

**Results:**

Vocal similarity between-speakers increased with a larger group size which indicates a higher cooperative vocal behavior. At the same time, there was an increase in EER for the same speakers between the smaller and the larger group size, meaning a decrease in overall recognition performance.

**Discussion:**

The decrease in vocal individualization in the larger group size suggests that ingroup cooperation and social cohesion conveyed through acoustic convergence have priority over individualization in larger groups of unacquainted speakers.

## 1. Introduction

Compared to other species, humans have an unparalleled ability to cooperate with unrelated individuals (McClung et al., 2017). The tendency to cooperate with others, however, is highly variable and dynamic (McClung et al., 2018), with ingroup membership and similarity promoting cooperation (Balliet et al., 2014). In this study, we approached the question of variation in human cooperation from an acoustic point of view. We modulated the parameter of group size to test (a) to what extent interlocutors in larger groups privilege individualization over cooperative accommodation when in need to be recognized, and (b) what the effect of either strategy, i.e., individualization or cooperation, is on voice discriminability in larger groups.

### 1.1. Vocal cooperation and its effect on voice individuality

How can individuals express cooperation in speech communication? During social interactions, cooperation typically manifests itself through convergent accommodation, i.e., the tendency of individuals to adjust aspects of their verbal and nonverbal behaviour towards those of their interlocutors in communicative encounters (for a recent overview, see a.o. Pardo et al., 2022). As posited by the Communication Accommodation Theory (Giles et al., 1991), accommodation can work in three directions: (a) convergence, which implies that speakers change their verbal and nonverbal behavior to become more similar, (b) divergence, implying that speakers apply modulations to become less similar and (c) maintenance, implying that speakers do not change during interaction. Experimental evidence revealed that speakers typically converge to signal closeness and sense of belonging to the same social group, to obtain social approval, to increase personal and social liking, as well as to regulate comprehension (Gallois et al., 2005). On the contrary, speakers diverge or maintain their communicative behavior when they wish to display valued social or ideological distinctiveness from others (Giles and Ogay, 2007) or regulate an extreme speech pattern of the dialogue partner (Pardo et al., 2022). Convergence and divergence have also been linked to task success and learning gain as documented in teamwork and human-computer interaction research. Convergence indeed was found to be more prevalent in higher than lower scoring teams and to be positively correlated with convergence toward spoken tutor dialogue systems (Friedberg et al., 2012; Thomason et al., 2013).

Accommodation is multidimensional. Evidence of convergence has been found in various linguistic and extra-linguistic features [for *syntax*, see a.o. (Branigan et al., 2000); for *lexicon* (Bell, 2002); *laughter* (Ludusan and Wagner, 2022), *facial expressions* (Lakin, 2013), and *body movements* (Dijksterhuis and Bargh, 2001)]. When it comes to vocal convergence, despite substantial inter-and intra-speaker variability, acoustic and perceived adjustments between conversational partners or between model talkers and shadowers, have been documented in numerous suprasegmental and segmental features, including *speech and pause rate, utterance duration, fundamental frequency, long-term average spectra*, *Mel-frequency cepstral coefficients*, *voice quality*, *voice onset time*, *vowel formants, clicks*, *utterance duration*, *amplitude envelopes*, *voicing contrasts* [see Pardo et al., 2022 for recent findings on vocal convergence; *cf.* Ostrand and Chodroff, 2021 for a study on holistic and individual measures of convergence].

While phonetic convergence is a good indicator of cooperation, social cohesion and proximity between communication partners, various types of evidence suggest that increased acoustic similarity between interlocutors’ phonetic repertoires may interfere with their auditory recognizability. In entertainment environments, for example, professional impersonators successfully pretend to be a target person by imitating, sometimes exaggerating, some of their vocal features. In forensic speaker comparison scenarios, voice imitation, a type of voice disguise used by perpetrators to conceal their real identity, increases the complexity drawing a conclucion whether two samples originate from the same speaker or different speakers (Eriksson, 2010). Empirically, it has been shown that exaggerated forms of convergence induced by intentional imitation of a voice or by voice conversion algorithms lead to a high error discrimination rate in automatic speaker recognition systems (Farrús et al., 2010). A similar effect has been found for human listeners, for which replicas and caricatures of target voices were shown to be perceived as similar and identical to the corresponding target voices, respectively, (López et al., 2013). Here, we posit that increased acoustic similarity may compromise individual recognizability. This suggests that constraints to human cooperation and thus to vocal convergence may arise in situations wherein auditory vocal recognition is at stake. Similar constraints have been shown, for example, in common marmoset vocalizations (Zürcher et al., 2021). Research examining the trade-off between vocal accommodation and individual recognizability in this species has found that the extent to which common marmosets converge in their vocal calls is highly influenced by the call type. Close contact calls happening with visual contact of conspecifics were observed to trigger more convergence than long distance calls produced without visual contact. This suggests that in the absence of visual cues to the identity of conspecifics, the vocal cues becomes essential for individual recognizability, and this in turn limits convergence.

Here we propose an experimental design to study the trade-off between vocal accommodation and voice individualization in humans. One option to carry out this task might have been to replicate the communicative scenario tested in marmoset communication, i.e., short versus long distance communication. We, however, refrained from replicating such a setting because of the known effects of talker-to-listener distance (henceforth TLD) on the variation of speech acoustics. It has been repeatedly shown that talkers spontaneously adjust their way of speaking in response to the increasing distance from the listeners, with consequences on various acoustic parameters, such as duration, intensity, fundamental frequency (f0) and formant frequencies (see a.o., Cheyne et al., 2009; Fux et al., 2011; Pelegrín-García et al., 2011). In this design, it would be hard to disentangle acoustic variations due to TLD and those to individualization or cooperation. A more promising alternative to modulating TLD has been found in group size. Inspired by findings on animal communication showing higher amount of vocal individuality in species living in larger groups (Pollard and Blumstein, 2011), and within the same species as the social network size increases (Mathevon, 2022), we designed a game-based communicative scenario wherein players in groups of different sizes auditorily recognized each other while playing a cooperative game (*cf.* par. The game design; par. 2.2. for the procedure). We modulated the parameter of group size to test the hypotheses that (1) individualisation increases with increasing group size of communication partners; (2) higher individualization in larger groups leads to better voice recognition. Given that divergence or maintenance are strategies to emphasize distinctiveness from others (Giles and Ogay, 2007), we expect that the acoustic similarity between the players decreases or remains stable from a smaller to a larger group. Nevertheless, in view of mixed evidence on the effect of group size on cooperation (see a.o. Wu et al., 2020), we cannot fully exclude that players may converge rather than diverge in larger groups. It has been shown, indeed, that the strategic situation as well as the individual and group payoff resulting from each member’s behaviour can influence cooperation in interactions (Capraro and Barcelo, 2015). For our current experiment, this implies that players’ incentive to converge or diverge in a larger group may vary according to the perceived cost *vs* gains associated with cooperation. Recalling the psychological factors through which groups size can affect cooperation [(1) expected others’ cooperation; (2) perceived collective efficacy; (3) perceived conflict of interest, Wu et al., 2020] it seems conceivable that players may converge if they prioritize group cohesion and affiliation, collective effort to complete the game over competing against each other in the interest of being recognized. If this should be the case, cooperative accommodation (convergence) prevails over individualization in larger groups and voices from individuals are better recognizable when they are obtained from interaction in smaller group as compared to larger group.

Which feature(s) can be used to study the effect of group size on individuals’ propensity to cooperate or enhance individuality? Accommodation and vocal individuality have been measured through a wide variety of spectral and temporal features with considerable inconsistency across studies, depending on the particular feature or set of acoustic features under examination (see a.o. Schweinberger et al., 2014; Pardo et al., 2022). Both phenomena are indeed multidimensional and speaker-specific.

Among the multitude of spectral and temporal features cueing individuality and accomodation, here, we used Mel Frequency Cepstral Coefficients (MFCC) as a numeric acoustic representation of speech information that is highly salient to humans (Davis and Mermelstein, 1980). MFCCs are the result of a series of signal processing techniques which turn a continuous spectral envelope into an underlying set of about 13–15 numbers, representing salient acoustic information like the fundamental spectral envelope shape of speech, including formants. This set of numbers is obtained from short windows of speech (~25 ms) at intervals of 10 ms. For each window, two fundamental processes are applied in the calculation of MFCCs, (a) obtaining values from the frequency axis through Mel filters in analogy to place-coding in the human cochlea and (b) obtaining regularities in the pattern of frequency-domain values through a Fourier analysis (Cepstrum; in MFCCs carried out by a cosine-transform). The resulting vector of 13 MFCC coefficients at 10 ms intervals is an acoustic representation that led to highly successful performance rates in a wide spectrum of speech and voice technology systems. They also contain a high amount of information about the acoustic individuality of the speech signal. To obtain a good representation of speaker-specific information, the signal needs to be further processed. Here we have chosen to use a common procedure which are fixed-length identity vectors (i-vectors; Dehak et al., 2011), obtained from the software VOCALIZE (Alexander et al., 2016). I-vectors reduce the 13xN-frames dimensional MFCC vector obtained from speech to a 400-dimensional vector as an acoustic model of a speaker’s voice. This is done by finding a fixed number of clusters in the 13-dimensional MFCC space through Gaussian Mixtures and reducing the clusters *via* some statistical processes that de-correlate the obtained information.

To understand whether humans would carry out individualization over convergent accommodation with increasing group size, we calculated the acoustic distances of their i-vector representations (henceforth speaker A, speaker B and speaker C) when playing in groups of 3 and 5 players, as well as within-speaker acoustic variability in in-game and post-game sessions. The similarity between i-vector representations was obtained in Vocalize *via* probabilistic linear discriminant analysis (PLDA), a statistical method for maximizing between-speaker and minimizing within-speaker differences.

### 1.2. Hypotheses

The two alternative hypotheses mentioned above concerning the effect of group size on cooperation vs. individualization (Aim 1) are more precisely reformulated in relation to PLDA scoring:

*Hypothesis 1a*: Individualization, acoustically manifested through divergence or maintenance, is expected to be more prevalent, if players' incentive to maximize their recognizability in larger groups prevails over cooperation. Hence, the average PLDA scores for between-speaker comparisons were expected to decrease from group *N* = 3 to *N* = 5 (henceforth: group 3 and group 5 respectively). *Hypothesis 1a* is henceforth referred to as the individualization hypothesis.

*Hypothesis 1b*: Cooperation, acoustically manifested through convergence, is expected to prevail over individualization in a larger group, if players prioritize group cohesion and affiliation as well as collective effort to complete the game over competing against each other in the interest of being recognized. Acoustically, we expected that the average PLDA scores for between-speaker comparisons would be higher when samples were obtained from group 5 than from group 3. *Hypothesis 1b* is henceforth referred to as the cooperation hypothesis.

Regarding within-speaker acoustic variability between in-game and post-game recordings, we predicted lower variability in post-game recordings, taken in isolation, as compared to in-game sessions.

Between the two game sessions, the individualization hypothesis would be confirmed if lower variability is found in group 5 compared to 3. On the other hand, the cooperative hypothesis would be supported if higher variability is found in recordings obtained in group 5 for the effect of the higher influence players exert on each other.

To examine the effect of individualization or convergence on voice recognition (Aim 2), we tested the speaker verification performance of the iVector/PLDA VOCALIZE system when post-game recordings were compared to the corresponding in-game recordings in group 3 and 5, in terms of Equal Error Rate (EER) using the software Biometrics Version 2019A. EER is a metric for a recognition system performance, corresponding to equal miss and false alarm rate. The lower the EER, the higher the recognition performance of the system. If the cooperation hypothesis holds (i.e., more cooperation in the larger group), we expected the EER to be higher when post-game recordings are compared to in-game recordings in group 5 than in group 3. Alternatively, if the individualization hypothesis is tenable (i.e., more individualization in the larger group), EER is lower when post-game recordings are compared to in-game recordings in group 5 compared to group 3.

The results of this study about the constraints to cooperative accommodation are relevant from an evolutionary perspective since they will shed further light on whether natural selection has also endowed humans (not only animal species) with the flexibility to privilege vocal accommodation or individuality depending on communicative circumstances. From a socio-psychological point of view, the results will contribute to a further understanding of human variability in cooperative behavior and team-building dynamics without visual cues. Additionally, with this research we will also collect further evidence about the implications of phonetic convergence on voice processing.

## 2. Materials and methods

### 2.1. The game design

Game-based techniques have been proved to be successful in eliciting semi-spontaneous (yet somewhat controlled) speech, as well as studying the suprasegmental properties of speech (Buxó-Lugo et al., 2018) and in facilitating phonetic convergence (Biro et al., 2022). In this study, we extend these findings to examine the trade-off between vocal accommodation and voice individualization as a function of group size. We designed a ludic cooperative situation wherein the players’ need to distinguish themselves varied. We invited participants in groups of different sizes to play a dominoes game online and to recognize each other during the match (§2.2 for the procedure). The interactive part of the game was designed based on findings on the factors affecting vocal accommodation. Decisions about the linguistic contents of the exchanges and the size of the groups were informed by empirical findings on factors affecting voice recognition and the design of voice memory/recognition tests (see below).

#### 2.1.1. Factors affecting vocal accommodation

Research on accommodation indicated that numerous factors can affect the degree and direction of interspeaker acoustic adjustments, including *time course of the conversation* (de Looze et al., 2014; Tobin, 2022), *conversational roles* (Pardo, 2006; Pardo et al., 2010), *speakers’ sex* (Namy et al., 2002; Weise et al., 2020), *frequency characteristics of lexical items and previous exposure* (Goldinger, 1998; Goldinger and Azuma, 2004), *task difficulty* (Abel and Babel, 2017), *task engagement* (Biro et al., 2022), *instructions to attend to partners’ speech* (Tausczik and Pennebaker, 2013), *visual attractiveness* (Michalsky and Schoormann, 2021), *likability* as well as *conversational quality* (Schweitzer et al., 2017; Michalsky et al., 2018). When convergence has been examined in conversational tasks, it has been shown that subjects do not remain involved to the same degree over the whole course of a conversation (Edlund et al., 2009; de Looze et al., 2014; Tobin, 2022). Information givers tend to converge more than information receivers (Pardo, 2006; Pardo et al., 2010), with male sex pairs converged to a greater or the same extent than female sex pairs (Pardo, 2006; Pardo et al., 2010; for alternative results, *cf.* Pardo et al., 2018). When examined in shadowing tasks, the linguistic characteristics of lexical items influenced convergence, with low-frequency words and previously heard lexical items evoking more convergence between shadowers and model talkers (Goldinger, 1998; Goldinger and Azuma, 2004). With this information in mind, in our experiment, all participants acted both as information givers and receivers, were all of the same sex (i.e., female), and they all repeated a script that linguistically varied only in number (from one to six) and color terms (blue, red, green, yellow, brown) (see par. 2.3. Speech Material). Conscious that excessively demanding tasks may drive the attention away from partners’ speech (Abel and Babel, 2017) and that engaging task environments favor convergence (Biro et al., 2022) in our experiment, participants were invited to play Dominoes, a renowned game with accessible mechanics in online interactive playrooms that permitted synchronized movements and real-time interactions between players through video conferencing tools.^1^ The reward system and game mechanics were designed to encourage players to make their voices easily recognizable, as one of the key criteria for winning was being the player best recognized. Additionally, players were encouraged to listen closely to the voices of their fellow players, as a separate reward was offered to those who achieved the highest recognition rates. With such a design we provide insights into whether acoustic individualization is a consciously employed strategy or whether the natural tendency towards convergence is stronger.

#### 2.1.2. Factors affecting voice recognition

Likewise accommodation, also voice recognition abilities are influenced by a wide range of stimulus-, speaker- and listener-related factors, including *duration and phonetic richness of the stimuli* (Sumby and Pollack, 1954; Bricker and Pruzansky, 1966; Roebuck and Wilding, 1993), *length of retention intervals* (see a.o. Legge et al., 1984; Kim et al., 2019), *size, quality and expressiveness of voice samples* (Legge et al., 1984; Yarmey et al., 2001; Kilgore and Chignell, 2006; Bregman and Creel, 2014; Bartle and Dellwo, 2015; Kim et al., 2019; Lavan et al., 2019; Zäske et al., 2020; Plante-Hébert et al., 2021), *context in which voices were learned* (Kim et al., 2019) as well as listeners’ *language ability* (Perrachione et al., 2011).

The findings related to the stimulus’ characteristics and the size of voice samples were of particular importance to the design of our game. Research showed that recognition is typically enhanced by longer stimuli, containing larger samples of the speaker’s phonetic repertoire (Sumby and Pollack, 1954; Bricker and Pruzansky, 1966; Roebuck and Wilding, 1993). With these premises, we controlled for the length of exposure to each speaker’s. As our primary interest is in voice modulations towards convergence or individualization, we asked the players to utter a 27–29 word-long carrier text that varied only in the number and color of dots on the dominoes, to avoid any confounding effects of idiosyncratic lexical choices on speaker recognition (see 2.3. Speech Material).We based the decisions concerning the size of groups on findings showing that 4–6 is the number of voices that (1) listeners can recognize with ease and short training (Legge et al., 1984; Yohan and Sommers, 2000; Kilgore and Chignell, 2006), (2) are used in the encoding phase of renowned voice memory/recognition tests (4 voices in the Glasgow Voice Memory Test: Aglieri et al., 2017; 4 voices in the Greenwich Voice Recognition Test Jenkins et al., 2021; 6 voices in the Jena Voice Recognition Humble et al., 2022). Therefore, we recruited one group of three and one group of five players for this experiment assuming that recognizing 2 to 4 speakers would not be particularly challenging. The recognition had to be done in the absence of visual cues with little familiarization and a non-canonical distinction between training and testing (see 2.2 Procedure).

### 2.2. Participants

Participants were five female Bernese German speakers aged between 22 to 26 y.o. (mean 24.5). Participants declared they had no known vision, no hearing, or linguistic impairment. To minimize the effect of idiosyncratic segmental realizations on voice recognition, all were auditorily pre-screened for comparable pronunciation patterns by a trained phonetician. The focus was on segmental features since they were shown to be used more than suprasegmental features in Swiss German dialect recognition (Leemann et al., 2018). Therefore, we aimed to control that speakers use the same variety of Bernese Swiss German to avoid that listeners may pick up on such segmental dialectal idiosyncrasies to recognize the speakers. To avoid the influence of an additional female voice on the players’ acoustic behavior, a male Bernese German speaker (22 y.o.) acted as the experimenter. Participants gave their informed consent to participate in the study and received monetary compensation for their participation. The study was conducted within the guidelines of the Ethics Committee of the Zürich University Faculty of Arts and Social Sciences.

### 2.3. Procedure

The experiment consisted of three main sessions: training, in-game, and post-game sessions.

**Figure 1:**
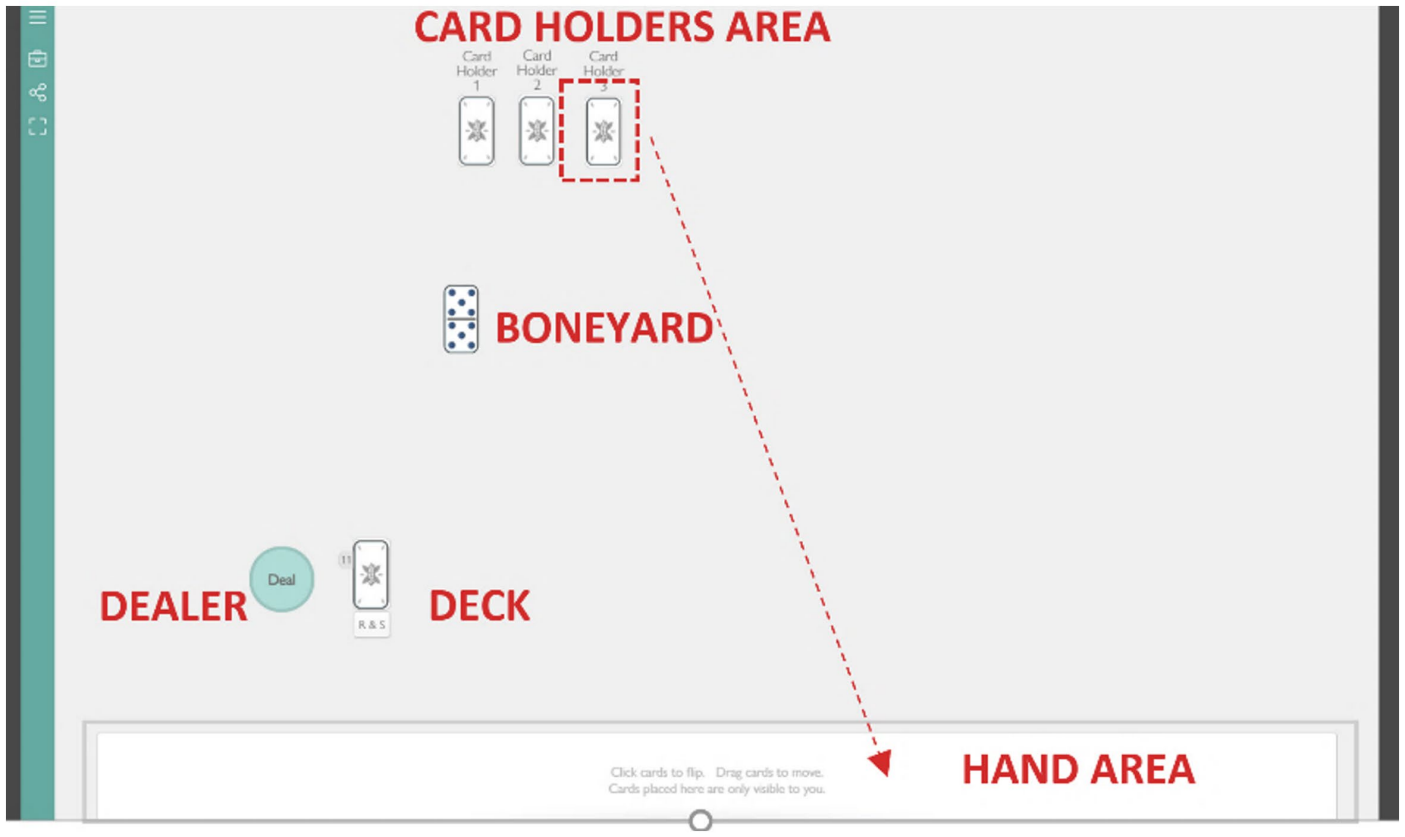
An example of the online Dominoes playroom (adapted from see footnote 1).

*Training*: One month before the start of the experimental session, every player received the game instructions *via* email and contacted the experimenter when they felt ready to start the training. During the training, individual players got familiar with the spaces of the online playroom and practiced the game mechanics with the experimenter (see Figure 1 for an example of the online dominoes playroom). After the training sessions, participants received a Zoom invitation link to the game session(s), an identification name (a letter from A to E), a player number (from 1 to 5), and a cardholder number (from 1 to 5). The Zoom identification name was unique for each player. Player’s and cardholder’s numbers varied between two game sessions to avoid the old players (from group 3) to have an advantage in attributing labels to voices.

*In-game sessions*: On the day of the experiment, the players joined the Zoom meeting with video off, the experimenter recalled the game rules, shared the link to the Domino playroom *via* the chat, and invited players to unmute their microphones and join the playroom. The first session was played by three players, and the second session by five (three from the first session plus two new players). The game mechanics for both game sessions are summarized as follows:

· All players move the first card from the cardholder to the hand area. Cards in the hand area are not visible to other players (step 1).

First round of the game:

· The player whose first card has the same number of dots as the one in the boneyard starts the game (step 2).
· The player greets (step 3) (e.g., Hello), reveals her identity (e.g., I am player one) (step 4), says what is on the card she has and the one she is looking for (e.g., I have the dominoes stone with six yellow tiles, I am looking for the dominoes stone with three red dots) (step 5). This procedure repeats until all players have revealed their identities (Figure 1).

**Figure 2:**
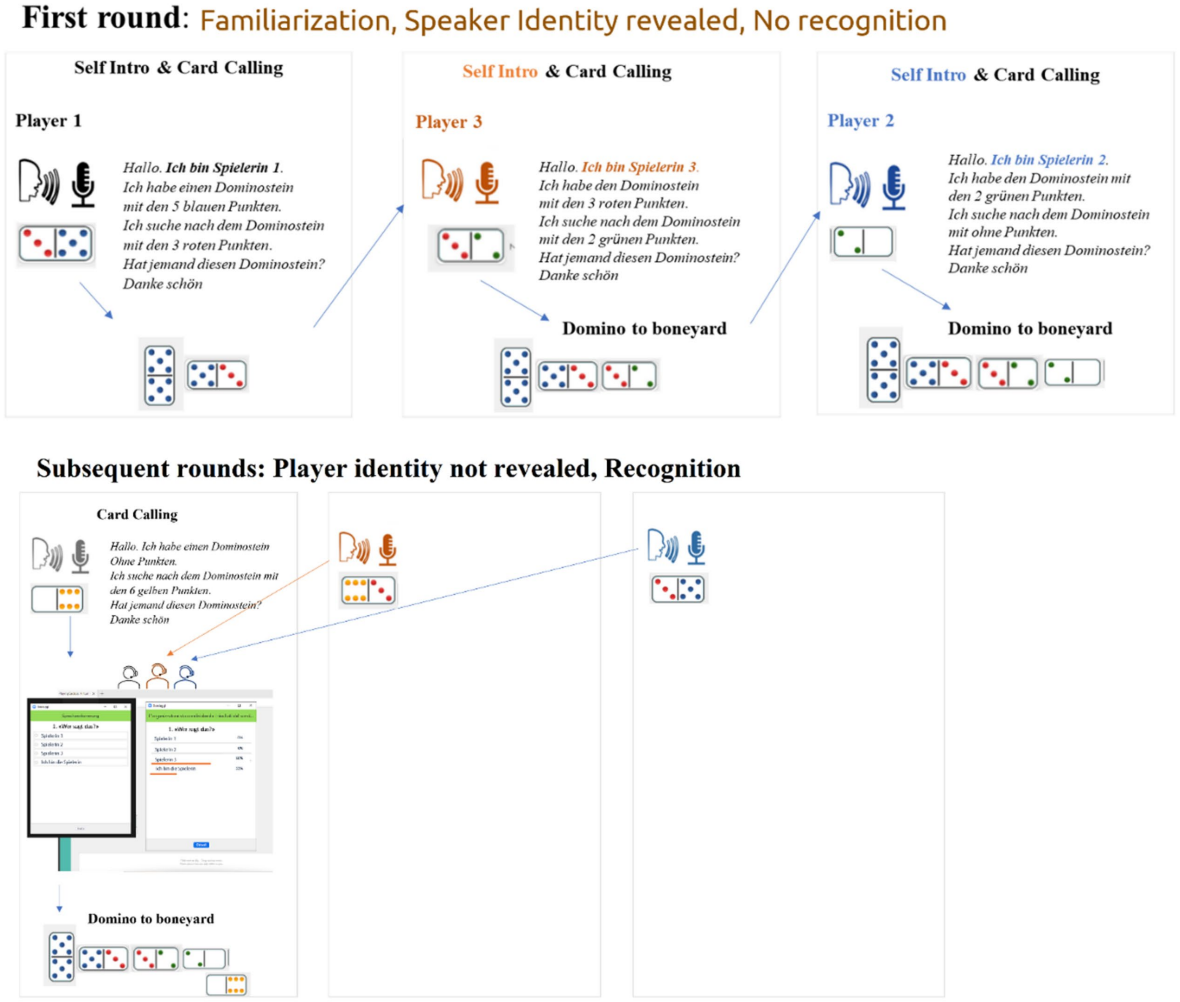
Illustration of the game mechanics for both the initial round (top) and subsequent rounds (bottom). In the first round, players reveal their identities by introducing themselves, which sets it apart from the following rounds. Starting from the second round onwards, the introductory stage is omitted, and instead, the experimenter administers a recognition test after each player’s turn.

From the second round onwards:

· The player who has a card matching the number of dots as the card in the boneyard starts the new round of the game (step 2), greets the participants (step 3) but does not reveal her identity (step 4 omitted). She says what is on the card she has and the one she is looking for (step 5) and after that the experimenter launches the Zoom poll (step 6). All players guess the identity of the speaker holding the turn. The player holding the turn clicks on the option “I am the player” (step 7).
· Once all players have voted, the experimenter shares the results of the poll (step 8). No correct answer is given, but it will be shown how often each given option was voted.
· A new player continues the game and the procedure repeats until all players have finished their cards. Step 4 happens only in the first round of the game (familiarization), while Steps 6, 7 and 8 from the second round onwards. The intersession interval was of about 2 h. Figure 2 displays the game mechanics for familiarization and the first post-familiarization round.

*Post-game sessions*: One month after the in-game sessions, the five players were invited again to record the utterances they had produced during the match (in group 5 only, or in groups 3 and 5) in single Zoom sessions. We took this set of recordings as a baseline since the players’ performance could not be influenced either by the acoustic behavior of the other players or by the need to be recognized.

The experimenter’s role in both the in-game and post-game recordings was consistent and limited to recalling the game instructions and marking the transition between different game rounds. He did not actively participate in playing the game. In in-game and post-game sessions, the speech performance was audio-recorded *via* Zoom on the local computer of the experimenter. Participants used their in-built microphones and headphones when playing the game. The option “Record a separate audio file for each participant” from the Zoom recording setting was enabled to obtain all participants’ audio streams as separate audio files. Every participant’s audio tracks from in-game and post-game sessions were converted from their original format (.m4a) to .wav format using Adobe Audition 2021 (32,000 samples/s and 16 bit-quantization).

### 2.4. Speech material

The speech corpus comprises 80 recordings of a 27–29-word long carrier texts in Bernese German, transcribed according to Dieth’s spelling (Dieth, 1986). An example of the game’s script in Bernese German and its relative translation in English is provided in (1). The underlined text was spoken only in the familiarization phase. Text varied between speakers and game rounds:

(1) Hallo. I bi d Spilerin 3. I ha dr Dominostei mit dä 3 rotä Pünkt. I sueche nachem Dominostei mit dä 2 grüene Pünkt. Het öpper dä Dominostei? Merci (*eng*. Hello. I am Player three. I have the dominoes card with three red tiles, and I am looking for the dominoes card with two green tiles. Who has this dominoes card? Thank you).

Of the 80 recordings, 15 were derived from the match in group 3 (3 speakers * 5 game rounds), 25 in group 5 (5 speakers * 5 game rounds), and 40 from post-game sessions. Of this latter set of recordings:

· 30 utterances for re-recording were derived from the three players playing in groups 3 and 5 [3 players * 10 rounds (5 rounds in group 3 + 5 rounds in group 5)].
· 10 utterances for re-recording were derived from the two players playing only in group 5 [2 players * 5 rounds].

Henceforth, we will refer to the speakers playing in both game sessions as Speaker A, Speaker B and Speaker C. Object of analysis for the present study are the utterances produced in-game and post-game sessions by Speak A, Speak B, and Speak C.

### 2.5. Features extraction, modelling and statistical analysis

For every dataset recording, 15-dimension MFCCs (Davis and Mermelstein, 1980) were calculated using a 32 ms Hamming windows with 50% overlap and 24 Mel filter banks in the range 1 Hz–4,000 Hz. The MFCC features were appended with delta and delta–delta coefficients, followed by Cepstral Mean Subtraction (CMS; Furui, 1981) which removes the convolutional noise. Further, the non-speech frames were dropped according to instantaneous-SNR-based voice activity detection (VAD; Kinnunen and Li, 2010). The processed MFCC features were used to extract 400-dimension i-Vectors (Dehak et al., 2011) from a pre-trained 1,024 component universal background model (UBM) and 400-dimension total variability matrix (TV) in the VOCALIZE software. Based on the recommendations in Kelly et al., 2019, the i-Vector speaker representations were not compared using a distance metrics, like co-sine similarity, but using a model-based comparison metrics, i.e., PLDA (Probabilistic Linear Discriminant Analysis), that is capable of using the most speaker discriminative parts from the i-vector speaker representation. PLDA scores for different and same speakers comparisons were obtained for speakers A, B and C for in-game recordings in groups 3, 5 and in post-game recordings.

To understand the effect of group size on players’ propensity to converge or diverge statistically, we first tested the effect of Groups (3 and 5) on PLDA scores for different speaker comparisons using Linear Mixed Effect Models. To account for speaker variability in accommodation behavior (Pardo et al., 2022) we entered the variable ‘speakers in comparisons’ (Speaker A *vs* Speaker B; Speaker A *vs* Speaker C and Speaker B *vs* Speaker C) as a random factor (i.e., random intercept). Given the evidence that during conversation, accommodation can evolve dynamically between turns (Edlund et al., 2009; de Looze et al., 2014; Tobin, 2022), we also entered the variable ‘game rounds’ (from 1 to 5) on which the PLDA score was calculated as a random factor (i.e., random intercept).

**Figure 3:**
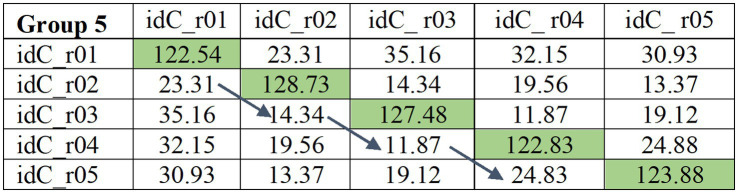
An example of PLDA matrix for same speaker comparison across game rounds. The blues arrow indicates the comparison of rounds on which ACD_PLDA was calculated.

To examine within-speaker variability in in-game and post-game sessions, we calculated the average consecutive difference PLDA (henceforth ACD_PLDA) for each speaker and game, in-game and post-game sessions (e.g., r01 compared to r02; r02 compared to r03, r03 compared to r04, r04 compared to r05, see Figure 3) according to the formula:


(1) $$ACD\_PLDA=\frac{\sum_{k=1}^{m-1}\left|Sk-Sk+1\right|}{m-1}\;$$

wherem=number of intervals;s=PLDA score of roundcom

Our hypothesis was that within-speaker variability would be lower in isolated post-game sessions compared to in-game sessions. In the latter, players’ acoustic behavior may be influenced by that of other players, leading to reduced within-speaker acoustic similarity across consecutive game rounds.

To test the effect of either individualization or naturally occurring convergence on voice discriminability in a larger as compared to a smaller group, we examined the speaker verification performance of the i-vector/PLDA system VOCALIZE when post-game recordings were compared to in-game recordings obtained in groups 3 and 5. The performance was measured in terms of Equal Error Rate (EER; Meuwly, 2000). EER was calculated from the PLDA scores between the extracted i-Vectors from the individual utterances produced by Speakers A, B and C when playing in groups 3 and 5 and in post-game recordings. The Biometric Version 2019A was also used for visualizing the system performance through EER plots.

## 3. Results

**Figure 4:**
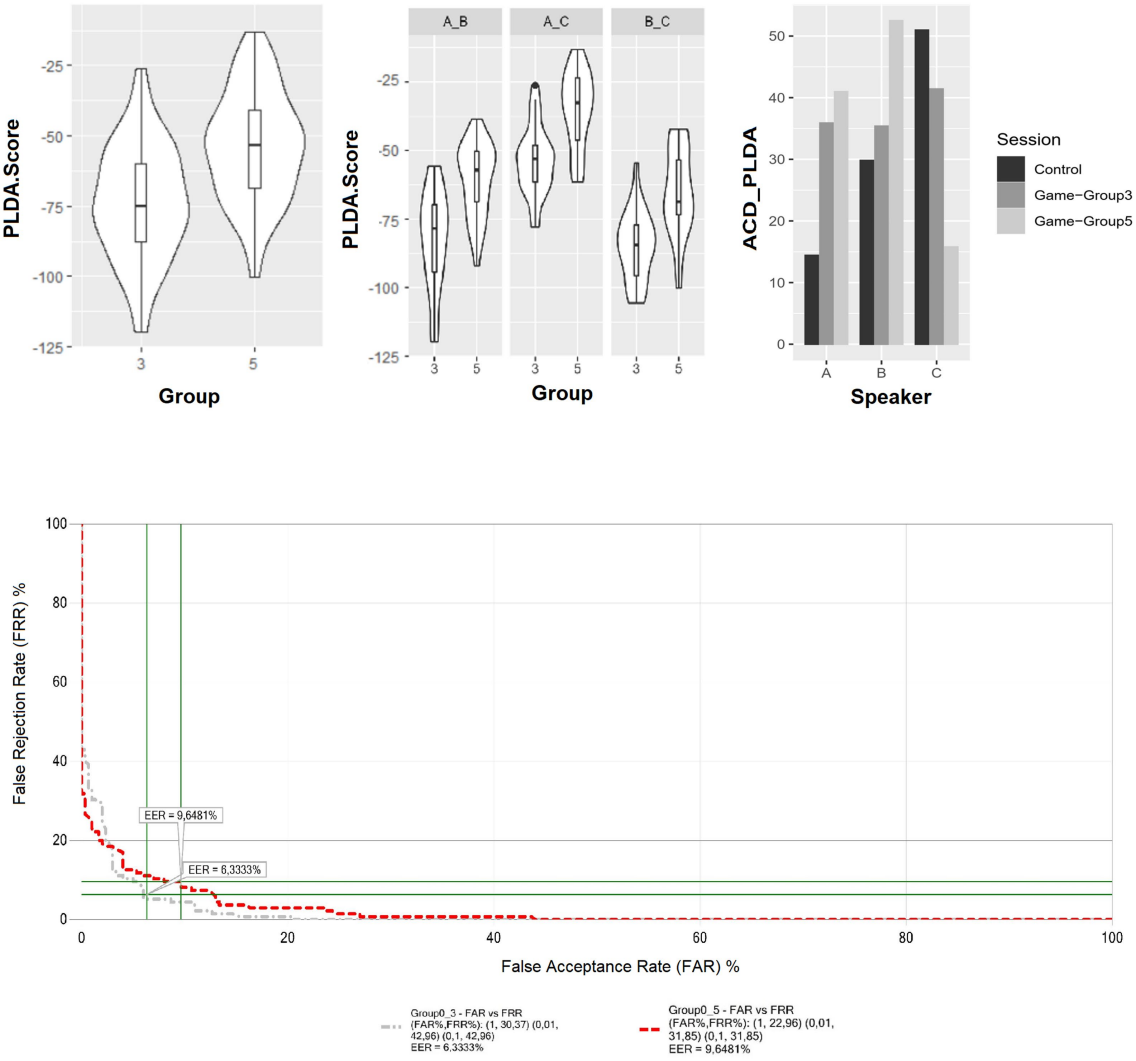
(Top left and center) PLDA scores for different speakers’ comparison per group (left), per group and speaker combination (centre). (Top right) ACD_PLDA scores for same speaker comparison by speaker and recording condition (group 3, group 5 and post-game recordings). (Bottom) EER Plots across group sizes. The grey line displays the false acceptance and false rejections rates when post-game recordings are compared with in-game recordings in group 3. The red line instead displays the same measures when post-game recordings are compared with in-game recordings in group 5.

Concerning aim 1, i.e., to understand whether humans would privilege individualization over convergent accommodation with increasing group size, the results of the statistical analysis showed a significant main effect of Group (5 compared to 3) on PLDA scores for between-speaker comparisons [*χ*^2^(1) = 61.19, *p* < 0.001]. Regarding the direction of the effect (e.g., cooperation vs. individualization), Figure 4 (top left) shows that PLDA scores in group 5 are higher than in group 3. This indicates that the similarity between the speakers increases in the larger group, hence supporting the cooperative cooperation hypothesis. The cooperation effect of playing in a larger group on vocal similarity is further validated when observing the behavior of individual pairs. As shown in Figure 4 (centre), for all three pairs (e.g., Speaker A - Speaker B; Speaker A - Speaker C; Speaker B - Speaker C), the PLDA score is on average higher in group 5. The generalized trend to cooperate is confirmed by the insignificant interaction between Group Size and Pairs on PLDA scores, tested with Mixed Effect Models, with game round as a random factor (i.e., random intercept) [*χ*^2^(2) = 0.265, *p* = 0.875].

As for the effect of group size on within-speaker acoustic variability across the experiment sessions, Speaker A and B behaved according to the predictions of the cooperative hypothesis. As shown in Figure 4 (top right), the ACD_PLDA in post-game recordings is lower than in in-game sessions, and between the two in-game sessions, the PVI_PLDA is higher in group 5 than in group 3. This suggests that within-speaker acoustic variability is the lowest in post-game recordings and the highest in recordings in the larger group, albeit to different extents between the three speakers. In Speaker C, instead, the lowest ACD_PLDA score in group 5 and highest in the post-game recordings support the individualization hypothesis: Speaker C is much more consistent in the degree of acoustic similarity when playing in group 5 compared to when speaking in isolation.

Concerning aim 2, i.e., to understand the effect of individualization or convergence on the verification performance of the automatic recognition system VOCALIZE, the results show that Equal Error Rate (EER) is larger when post-game recordings are compared to in-game recordings in group 5 (9.64%) than to those in group 3 (6.33%) (Figure 4 bottom). The EER is inversely related to the accuracy of the biometric system. Therefore, the results indicate a decrease in the system’s performance when the comparison involved in-game recordings in group 5, characterized by inter-speaker acoustic cooperation. This suggests that acoustic cooperation comes at the cost of reduced individuality in the acoustic structure of speech, with a negative impact on individual speakers’ recognizability.

## 4. Discussion

In the current study, we introduced a novel game-based method to investigate the trade-off between cooperative accommodation and voice individualization in situations wherein voice recognition was at stake. Inspired by findings from animal communication (Pollard and Blumstein, 2011; Mathevon, 2022) and human social sciences (Capraro and Barcelo, 2015; Wu et al., 2020), we tested two alternative hypotheses – individualization and cooperative hypotheses - about the effect of group size on human vocal modulations and recognizability. Overall results showed that (a) between-speaker similarity increases with a larger group size, (b) within-speaker variability is predominantly higher in in-game sessions than in post-game recordings, (c) the automatic system discrimination performance, measured in terms of EER, was higher when post-game recordings were compared with samples from group 5 compared to group 3.

What do these findings tell about the effect of group size on human vocal cooperation and individualization? Regarding the hypothesized impact of group size on players’ preference toward convergent accommodation *or* individualization, our results point in favor of the cooperation hypothesis. Unlike findings in animal communication showing that group size has a positive impact on the degree of individuality in vocalizations across taxa and within-species (Pollard and Blumstein, 2011; Mathevon, 2022) in the given experimental circumstances, it seems that interactions in larger groups of unacquainted speakers are the type of communicative settings in which signaling ingroup cooperation and social cohesion through acoustic convergence have priority over individualization.

**Figure 5:**
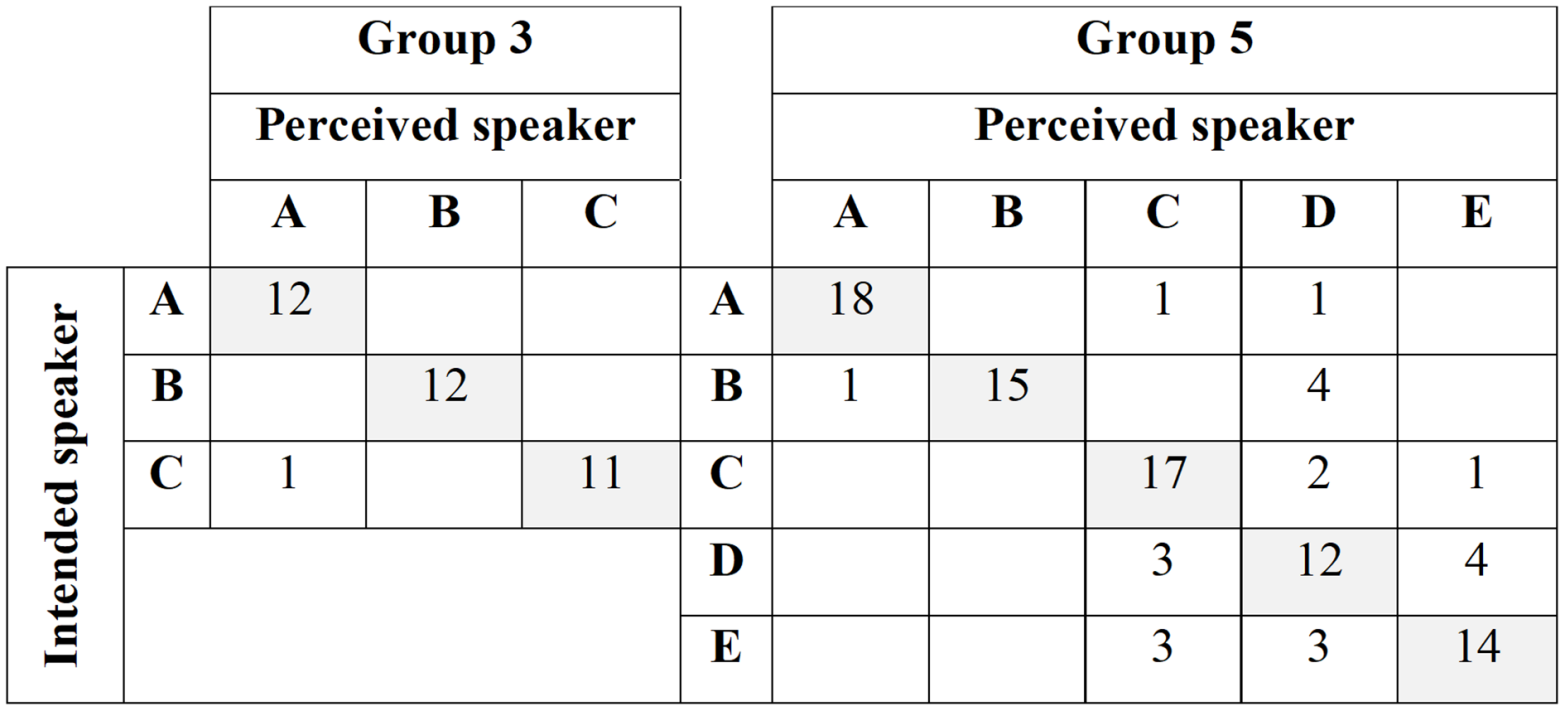
Confusion matrices about intended and perceived speakers across game sessions played in Group 3 and 5.

An alternative plausible explanation for the negative impact of group size on human vocal individualization considers the factors influencing human cooperation, i.e., perceived potential costs *vs* benefit of cooperation. As suggested by two of the most prominent theories of accommodation – Communication Accommodation Theory (Giles and Ogay, 2007) and Interactive Alignment Model (Pickering and Garrod, 2004) – between the two main accommodation directions, convergence is the default pattern that can also happen without specific conversational demands (Wagner et al., 2021). On the other hand, divergence is the marked pattern and is typically triggered when individuals have strong personal, social, or linguistic motivations to distance themselves from their interlocutors. In our study, the game dynamics were designed to ensure sufficient motivation for players to diverge and compete in larger groups to enhance their recognizability. However, the recognized costs of divergence must have been perceived to be relatively high compared to the benefit of maintaining the natural tendency to converge. This can be imputable to the recognition feedback provided to the players during the match. Given that players were satisfactorily recognized (Figure 5), we cannot exclude that what was supposed to be a trigger to vocal individualization might have turned into an incentive to converge. There is another explanation for the observed convergence that is not related to the group size (5 vs. 3) or the identity recognition task. It is possible that the voice convergence observed in group 5 is simply a result of speakers A, B, and C having a longer duration of exposure to one another over the course of two sessions. The two-hour interval between the game sessions may have not been sufficient to prevent long-term accommodation effects from occurring (reviewer 1, review communication).

Further testing is needed to validate the experimental method in a larger cohort and to confirm the observed pattern of convergence by including the traditional acoustic measure of convergence (Ostrand and Chodroff, 2021) and voice individuality (e.g., F0, harmonicity, vowel formants, formant dispersion, and speech rate; Latinus et al., 2013; Schweinberger et al., 2014). Different computational approaches need to be used to observe the direction of interspeaker adjustments (who converges to whom?). From the results on the acoustic convergence in group 5, it is not possible to infer whether the increased between-speaker similarity was achieved through mutual inter-speaker adjustments or by one speaker converging towards another. Based on the lower within-speaker variability in game sessions observed for Speaker C, here we speculate that accommodation was mainly driven by Speaker A and Speaker B adjusting their vocal features towards C. This hypothesis as well as the directionality of convergence between Speak A and B needs to be clarified. It then may shed further light on the different degree of adaptability interlocutors may have in conversations. Research shows that speakers vary in the way they accommodate for a multitude of reasons, and that individuals can even converge in one set of attributes for one set of speakers/− model talkers and another set of attributes for another set of speakers (Pardo et al., 2017). With these premises, it is thus possible that the observed pattern of convergence in MFCC-related features does not replicate when quantifying accommodation with other acoustic features extracted from longer time frames. For future research it would be interesting to examine individualized and cooperative features by individuals and pairs. Exploration of alternative methods is needed to reveal how the dynamics of convergence or individualization change over time, potentially shedding light on the underlying processes at play in the game.

Another finding of this study that supports the cooperative hypothesis is the poorer performance of the automatic recognition system VOCALIZE in samples from group 5 compared to post-game recordings. The negative impact of convergence on the system performance is not surprising and is in lines with previous research showing the vulnerability of voice-based authentication and recognition systems to deliberate forms of convergence through voice imitation or voice conversion algorithm (Farrús et al., 2010; Kinnunen et al., 2012). This suggests that not only exaggerated natural or artificial forms of vocal convergence but also spontaneously occurring forms of convergence can impact the performance of automatic speaker recognition systems.

Although additional acoustic investigations with changes in the game setup (e.g., providing recognition feedback at the end of the game session) are needed to confirm the observed prevalent cooperation in larger groups, the findings of this study support the importance of eliciting accommodation with engaging game-based techniques (Biro et al., 2022) but also motivate further investigations on human vocal flexibility between cooperation and individualization.

## Data availability statement

The raw data supporting the conclusions of this article will be made available by the authors, without undue reservation.

## Ethics statement

Participants gave their informed consent to participate in the study and were paid for their participation. The study was conducted within the guidelines of the Ethics Committee of the Zürich University Faculty of Arts and Social Sciences.

## Author contributions

EP and VD conceived and designed the experiment, revised the manuscript and discussed milestones. EP conducted the experiment, analysed the data and drafted the manuscript. All authors contributed to the article and approved the submitted version.

## Funding

This study was supported by the NCCR Evolving Language, Swiss National Science Foundation Agreement #51NF40_180888.

## Conflict of interest

The authors declare that the research was conducted in the absence of any commercial or financial relationships that could be construed as a potential conflict of interest.

## Publisher’s note

All claims expressed in this article are solely those of the authors and do not necessarily represent those of their affiliated organizations, or those of the publisher, the editors and the reviewers. Any product that may be evaluated in this article, or claim that may be made by its manufacturer, is not guaranteed or endorsed by the publisher.

## Footnotes

^1^ https://playingcards.io/
